# Characterization of bismuth-based photocatalyst for microcystin-LR degradation and mechanism: a critical review

**DOI:** 10.1098/rsos.241506

**Published:** 2025-05-28

**Authors:** Sakil Mahmud, Ning Zhang, K. V. Nedunuri Kumar

**Affiliations:** ^1^ Department of Water Resources Management, Central State University, Wilberforce, OH, USA; ^2^ Department of Chemistry and Physics, Lincoln University—Main Campus, Philadelphia, PA, USA

**Keywords:** bismuth photocatalyst, synthesis mechanism, microcystin degradation, photocatalysis of microcystin, microcystin degradation mechanism

## Abstract

The utilization of bismuth (Bi)-based photocatalysts for the degradation of microcystin-LR (MC-LR) has gained significant attention in recent years owing to their promising photocatalytic properties. This paper critically reviews the characterization and mechanisms of Bi-based photocatalysts for MC-LR degradation. Bi-based materials, particularly Bi oxyhalides and Bi oxides, have shown excellent photocatalytic performance in degrading MC-LR under visible-light irradiation. Various synthesis methods, including hydrothermal, solvothermal and template-assisted methods, have been discussed to tailor the morphology and properties of Bi-based photocatalysts for enhanced MC-LR degradation. The photocatalytic mechanisms involved in MC-LR degradation by Bi-based photocatalysts are elucidated, highlighting the roles of reactive oxygen species and electron–hole pairs in the degradation process. Additionally, the factors influencing the photocatalytic activity of Bi-based photocatalysts, such as crystal structure, surface area and doping, are discussed. Challenges and future prospects in developing and applying Bi-based photocatalysts for MC-LR degradation are also addressed, emphasizing the need for further research to optimize their performance for practical environmental remediation applications.

## 1. Introduction

Water pollution is a critical environmental concern worldwide, with various sources contributing to the contamination of water bodies [1]. One significant source of water pollution is cyanobacterial algal blooms, which have become increasingly prevalent because of factors such as nutrient enrichment from agricultural run-off, industrial discharges and urban wastewater [2–4]. These blooms can produce a range of toxins known as cyanotoxins, such as microcystins (MCs), posing serious threats to aquatic ecosystems, human health and the economy [5]. In response to this environmental challenge, researchers have been diligently seeking fast and efficient methods to control algae growth and meantime remove a variety of toxins [6,7]. Conventional approaches, including physical, chemical and biological methods, have shown limitations in terms of effectiveness and environmental impact [6,8]. Physical methods such as interception and filtration are quick but often costly and limited in scope [9]. Chemical methods, involving the use of chemical algae removers or coagulants, can lead to secondary pollution [10]. Biological methods, which use algae-inhibiting bacteria or allelopathic plants, are slow and prone to repetition [11].

**Figure 1.**
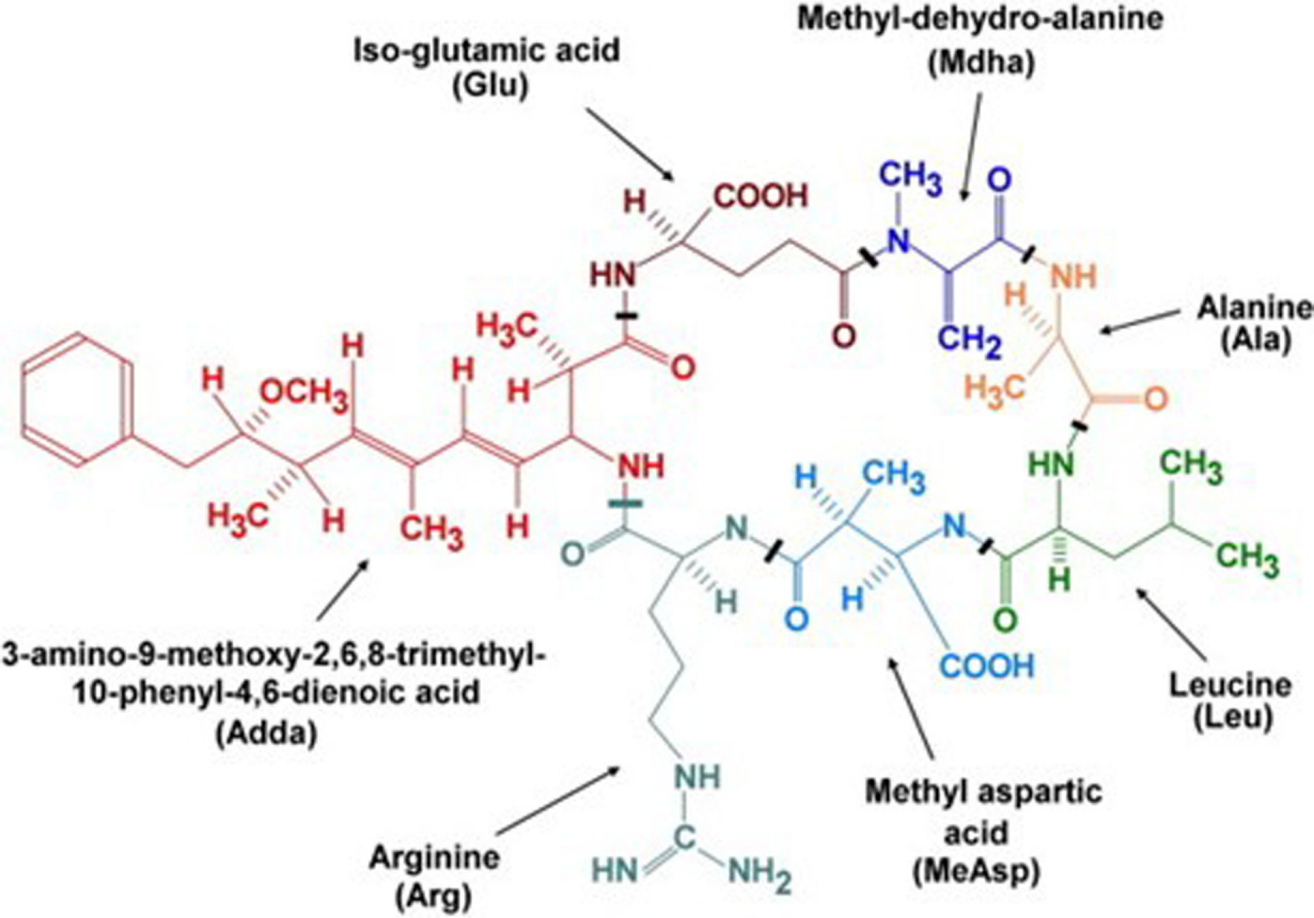
The structure of MC-LR [19]. Copyright 2008 Elsevier Ltd.

In recent years, photocatalytic technology has emerged as a promising approach for algae inhibition [12]. This technology uses semiconductors as photocatalysts to convert light energy into chemical energy, effectively harnessing electrons and holes generated during this energy conversion process to participate in oxidation-reduction reactions [13,14]. The use of nanomaterials in photocatalysis has further enhanced its efficacy, as defects and suspended bonds in these materials can capture electrons or holes, preventing their recombination and enhancing their redox potential [15]. This redox ability can effectively inhibit or kill algal cells and decompose cyanotoxins, making photocatalysis a potential solution for algal bloom control [16]. Among the cyanotoxins produced by cyanobacterial blooms, MCs are of particular concern owing to their widespread occurrence and life-threatening organ toxicity [17]. Microcystin-LR (MC-LR) is one of the widely detected variants of MCs, with a range of adverse health effects, including liver damage, carcinogenicity and neurotoxicity [18]. Molecular structure of MC-LR is shown in figure 1. MCs are intracellular toxins produced through metabolic activities. However, MCs can be released from cyanobacteria cells under stressful environmental conditions, such as increased temperature, depletion of nutrients, presence of heavy metals and stagnant water flows [20]. The World Health Organization has established a provisional guideline value of 1.0 µg l^−1^ for MC-LR in drinking water, underscoring the need for effective treatment methods to remove this toxin from water sources [21,22].

As mentioned, photocatalysis has emerged as a promising technology for the degradation of organic pollutants, including MC-LR, in water. Semiconductor photocatalysts, such as titanium dioxide (TiO_2_) [23] and zinc oxide (ZnO) [24], have been extensively studied for their ability to degrade organic contaminants under UV light irradiation [25,26]. However, their limited efficiency under visible light has prompted the search for alternative photocatalysts with improved visible-light response. Bismuth (Bi)-based photocatalysts have shown great potential as visible-light-activated photocatalysts for MC-LR degradation [27]. Bi oxyhalides, such as Bi oxyiodide (BiOI), Bi oxychloride (BiOCl) and Bi oxybromide (BiOBr), are among the most widely studied Bi-based photocatalysts for MC-LR degradation [28–30]. These photocatalysts exhibit favourable properties, including increased band gap, enhanced photocatalytic performance, visible-light response, stability and low toxicity, making them attractive for water treatment applications [30].

Bi-based photocatalysts possess unique properties that make them suitable for MC-LR degradation. Their critical characteristics behind MC-LR degradation can be noted as follows: (i) one of the key advantages of Bi-based photocatalysts is their ability to absorb visible light, which allows them to use a larger portion of the solar spectrum for MC-LR degradation under natural conditions [31,32]. This visible-light response is attributed to the electronic structure of Bi-based compounds [33], which differs from that of traditional photocatalysts such as TiO_2_ and ZnO. While TiO_2_ and ZnO have wide band gaps (around 3.2−3.3 eV) limiting them to UV light absorption, Bi-based compounds have narrower band gaps (2.0−2.8 eV), allowing for visible-light absorption. This is owing to the hybridization of Bi 6s and O 2p orbitals, reducing the band gap. Additionally, the strong spin–orbit coupling in Bi compounds enhances charge separation and reduces electron–hole recombination, making them more effective under visible light for applications like MC-LR degradation; (ii) the crystal structure of Bi-based photocatalysts plays a crucial role in determining their photocatalytic activity [34]. Bi oxyhalides typically exhibit a layered structure, with alternating layers of Bi-oxide and halide ions [35]. This layered structure provides a large surface area for photocatalytic reactions and facilitates the migration of charge carriers, leading to enhanced photocatalytic activity; (iii) doping is another strategy that can be used to enhance the photocatalytic activity of Bi-based photocatalysts [36]. The introduction of dopant atoms can modify the electronic structure of the photocatalyst, leading to improved charge separation and reduced recombination rates [37]. Doping with metals such as iron (Fe), nickel (Ni) and copper (Cu) has been shown to enhance the visible-light photocatalytic activity of Bi oxyhalides for MC-LR degradation [38]; and (iv) the morphology of Bi-based photocatalysts also plays a significant role in determining their photocatalytic activity [39]. Various morphologies, such as nanosheets, microspheres and hierarchical structures, have been synthesized and studied for their photocatalytic properties. These morphological features can affect the surface area, light absorption properties and charge carrier mobility of the photocatalyst, ultimately influencing its photocatalytic activity [40].

MC-LR degradation on Bi-based photocatalysts involves a series of complex chemical reactions [41]. Upon irradiation with visible light, Bi-based photocatalysts generate electron–hole pairs, which can then participate in redox reactions with MC-LR molecules adsorbed on the photocatalyst surface [42]. For example: (i) one of the key mechanisms of MC-LR degradation on Bi-based photocatalysts is the generation of radical species with strong oxidizing capability, such as hydroxyl radicals (·OH) and superoxide radicals (·O_2_^−^), that can decompose organic molecules, including MC-LR, and convert complicated molecules into smaller, less toxic compounds. The generation of reactive oxygen species (ROS) on Bi-based photocatalysts is facilitated by the presence of oxygen and water molecules in the reaction environment [43]; (ii) another important mechanism of MC-LR degradation on Bi-based photocatalysts is the direct oxidation of MC-LR by photogenerated holes [44]. The photogenerated holes can directly oxidize the electron-rich sites on the MC-LR molecule, leading to the cleavage of its chemical bonds and the formation of degradation products [27]; and (iii) the pH of the reaction solution can also influence the photocatalytic degradation of MC-LR on Bi-based photocatalysts [45]. The pH affects the surface charge of the photocatalyst and the protonation state of MC-LR, thereby influencing their interaction and degradation kinetics [46]. Generally, the degradation efficiency of MC-LR is more than 50% under acidic conditions owing to the enhanced generation of ROS [46].

The objective of this review is to provide a comprehensive overview of the characterization of Bi-based photocatalysts for MC-LR degradation and the underlying mechanisms involved. We will discuss the structural and chemical properties of Bi-based photocatalysts that contribute to their photocatalytic activity, as well as the factors influencing their performance. Additionally, we will examine the degradation pathways of MC-LR on Bi-based photocatalysts and the role of reactive species in the degradation process. Overall, this review aims to highlight the potential of Bi-based photocatalysts as effective and environmentally friendly materials for removing MC-LR from water sources. By understanding the mechanisms involved in MC-LR degradation on Bi-based photocatalysts, researchers can develop strategies to optimize their performance leading to their practical application in water treatment systems.

## 2. Synthesis of bismuth-photocatalysts

**Figure 2.**
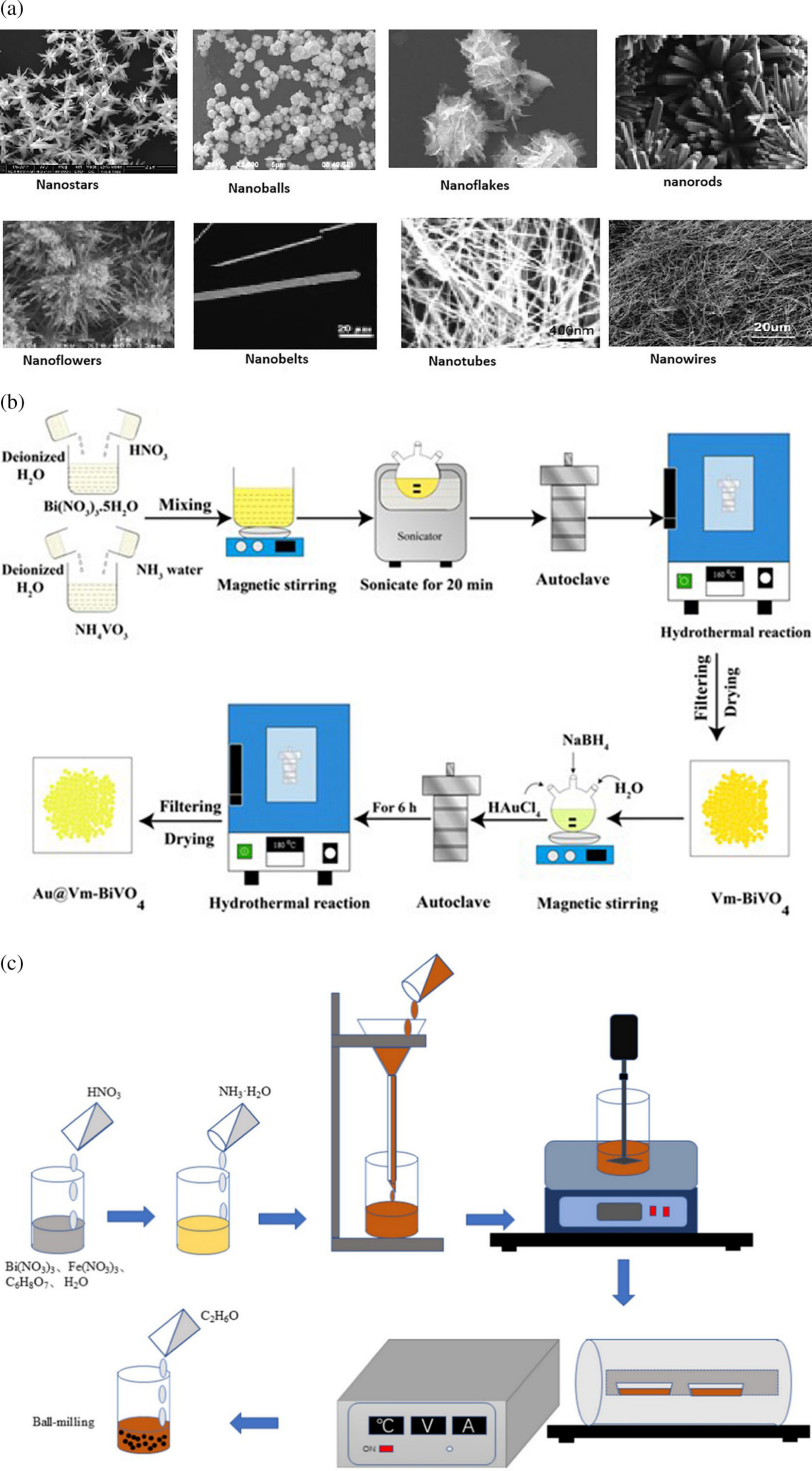
Schematic for the synthesis of bismuth-based photocatalysts: (a) hydrothermal method (reprinted with permission [59], Royal Society of Chemistry 2023); (b) the sol–gel technique [60]; and (c) examples of various nanostructures of bismuth nanoparticles (reprinted under the terms of CC BY [61], Elsevier 2021).

There are several techniques for synthesizing Bi-based photocatalysts, each with its advantages and applications [47]. Common techniques include hydrothermal/solvothermal synthesis [48,49], the sol–gel method [50], co-precipitation [51], ion exchange [52,53] and atomic layer deposition [54,55]. The choice of synthesis method depends on the desired properties and applications of the Bi-based photocatalyst [56]. Each method offers unique advantages regarding control over composition, structure and properties, allowing researchers to tailor the photocatalyst to meet specific application requirements [57,58]. Examples of Bi nanostructures include star-shaped, spherical, belt-like, ribbon-like, flower-like, snowflake-shaped, tubular, rod-like and wire-like formations, as illustrated in figure 2a.

### 2.1. Hydrothermal synthesis

Bi-based photocatalysts have gained significant attention owing to their potential in various applications, including environmental remediation and solar energy harvesting, where they convert solar energy into chemical energy for processes such as photocatalytic water splitting and solar fuel generation [62,63]. The hydrothermal method is one of the most used techniques for synthesizing Bi photocatalysts, offering control over particle size, morphology, surface area and crystallinity [64,65]. A schematic representation for synthesizing a Bi-based photocatalyst using the hydrothermal method is shown in figure 2b. This method involves reacting with precursor materials in a high-pressure, high-temperature aqueous solution [59,66]. For example, Zhu *et al*. [67] synthesized Bi ferrite (BiFeO_3_) nanocrystals with diameters ranging from 10 to 50 nm using a hydrothermal method at reduced temperatures. The use of surfactants and polymers in the hydrothermal process can influence the morphology and size of Bi photocatalysts. Dharmiah *et al*. [68] demonstrated the preparation of three different nanostructures of Bi telluride (Bi_2_Te_3_) using surfactants such as ethylenediamine tetraacetic acid (EDTA), polyvinylpyrrolidone (PVP) and ethylene glycol, resulting in nanoflakes of 300 nm, nanoplates of 200 nm and nanospheres of 25 nm, respectively [3]. Moreover, Bi-based photocatalysts synthesized by the hydrothermal method have been employed for specific applications. For example, Houlberg *et al*. [69] synthesized phase-pure Bi substituted with ceria (Bi-CeO_2_) using a hydrothermal method, demonstrating its potential for developing efficient photocatalysts in reducing organic pollutants. Dai *et al*. [70] reported the enhanced photocatalytic performance of the oxychloride/BiOI nanocomposite sheets fabricated by the hydrothermal method for dye degradation under visible light. Overall, the hydrothermal method, along with variations such as microwave-assisted hydrothermal synthesis and the introduction of surfactants and polymers, offers a versatile approach for synthesizing Bi-based photocatalysts with tailored properties for various applications [71,72].

### 2.2. Chemical reduction technique

The chemical reduction technique is a crucial method for synthesizing Bi-based photocatalysts, especially nanoparticles, for diverse applications like water and air purification [73–75]. It involves reducing Bi ions from a precursor solution using agents like sodium borohydride or hydrazine, leading to the formation of Bi nanoparticles [76,77]. This process requires precise control of parameters such as temperature and pH to ensure uniform nanoparticle size and shape. Stabilizing agents may be added to prevent agglomeration [78]. Polyvinylpyrrolidone (provides steric stabilization), citrate ions (form electrostatic stabilization), polyethene glycol (ensures steric stabilization to maintain uniform size), surfactants (form protective layers around nanoparticles), polyethyleneimine (provides both steric and electrostatic stabilization) and tannic acid (stabilizes through hydrogen bonding and electrostatic interactions) are common examples. After synthesis, nanoparticles are separated, purified and often subjected to drying and calcination to enhance their properties. Calcination, a thermal treatment process, is crucial for improving the crystallinity, removing organic residues and stabilizing the desired phase of the nanostructures. For example, calcination of Bi₂O₃ can transform amorphous or metastable phases into the highly active α-Bi₂O₃ phase, which exhibits superior photocatalytic performance. Additionally, calcination enhances the material’s surface area, porosity and thermal stability, all of which contribute to improved photocatalytic activity. Various chemical synthesis techniques, including polyol [79], electrochemical [80] and photochemical methods [81], are employed to prepare diverse Bi nanostructures [82]. The polyol technique, a chemical reduction method, offers advantages such as cost-effectiveness and simplicity. It involves solvating the metal precursor, reducing and nucleating the monomer agent and growing the nuclei to form nanoparticles [83]. For instance, Wang *et al*. controlled the size and shape of Bi nanoparticles using PVP as a capping polymer in ethylene glycol at 200°C, resulting in various nanostructures such as cubes and triangular plates [84]. Alcohols, hydrazine and citrate ions are robust reducing agents for Bi nanoparticle synthesis. Brown & Goforth synthesized Bi nanoparticles using hydrazine and observed increased stability when dextran was present in the solution [85]. Another method used borane as a reducing agent that was found to enhance the stability and payload of Bi nanoparticles and also improve their sensitivity when used as contrast agents in computed tomography scans. Electrochemical methods, including electrodeposition and photochemical techniques, are also employed for Bi nanoparticle synthesis. Zhao *et al*. [86] used a photo deposition method to prepare Bi sulfide nanoflowers over an alumina template, with thioacetamide as a sulfur source. The reaction time and solution pH significantly influenced the morphology of the synthesized nanoflowers, demonstrating the versatility of the photochemical approach. In summary, various synthesis methods offer precise control over the size, shape and properties of Bi nanoparticles, enabling their utilization in diverse applications ranging from catalysis to biomedical imaging.

### 2.3. Sol–gel technique

The sol–gel technique emerges as a sophisticated, wet chemical process enabling the synthesis of a broad spectrum of oxide materials, including multifaceted compositions like spinels and perovskites, without the necessity for high-temperature calcination or costly alkoxide reactants [87–89]. This method offers unparalleled control over the material’s surface architecture and homogeneity at significantly reduced temperatures compared to traditional methods. The process can be finely tuned by varying the conditions, such as the nature of the solvent, the initial materials, gelation time and environmental factors like pH and temperature, along with the introduction of surfactants or catalysts [90–92]. Through diverse approaches like electrospinning, template deposition, heating and spin coating applied to the sol–gel precursor, nanoparticles manifesting in shapes from nanowires and nanotubes to thin films and spheres can be achieved [93–95]. Figure 2c illustrates a schematic diagram depicting the synthesis process of Bi-based photocatalysts via the sol–gel technique.

Recent applications of the sol–gel method have been directed towards the fabrication of binary oxides, evidencing its versatility. Various research groups have demonstrated the synthesis of complex Bi-oxide-based nanocomposites, such as BiOCl–TiO_2_, Bi ferrite and Bi_2_O_3_–CuO alongside ZnO–SiO_2_, and even Bi germanate (Bi_4_Ge_3_O_12_), through this method [96–98]. The first reported synthesis of ultrafine Bi-oxide particles using the sol–gel technique involved reflux reactions to create a gel of Bi hydroxide, which was then allowed to crystallize [99]. The morphology and purity of the resultant Bi nanoparticles are significantly influenced by the choice of chelating agents, such as acetic acid, tartaric acid and citric acid, during the synthesis process, highlighting the importance of chelating agents in the successful preparation of Bi nanomaterials [93]. A notable study by Wang *et al*. [100] detailed the synthesis of pristine Bi iron oxide nanostructures with a polyhedral morphology and sizes ranging between 60 and 90 nm using tartaric acid as a chelating agent, illustrating the method’s efficacy in producing complex oxide nanostructures.

### 2.4. Sonochemical technique

**Figure 3.**
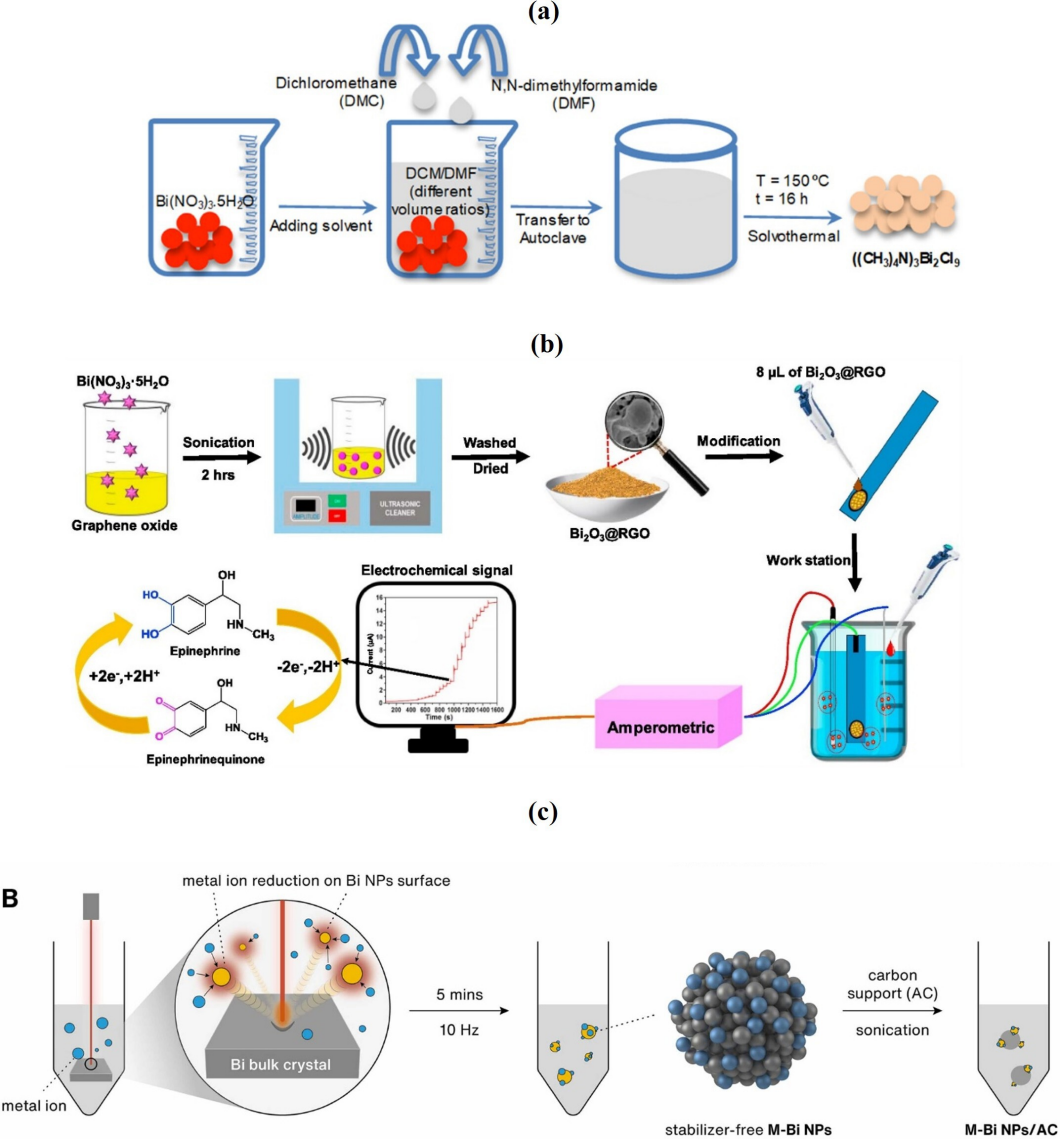
Schematic for synthesizing a bismuth-based catalyst: (a) solvothermal method (reprinted with permission [49], ACS, Springer 2023); (b) sonochemical technique (reprinted with permission [105], Elsevier 2019); and (c) laser-aided technique (reprinted with permission [106], Elsevier 2022).

The sonochemical technique presents a versatile and straightforward method for synthesizing diverse nanostructures, using the extreme conditions generated by ultrasound, such as pressures above 1000 atm and temperatures exceeding 5000 K [101]. This method harnesses two fundamental physical phenomena associated with ultrasound: acoustic cavitation [102], involving the implosive collapse of bubbles in the liquid, and nebulization [103], leading to mist formation as ultrasound passes through the liquid and interfaces with gas. Compared to traditional techniques, sonochemistry offers rapid reaction rates, facile reaction conditions and the ability to produce nanoparticles with uniform shapes, high purities and narrow size distributions [101,104]. A synthesis technique of a Bi-based catalyst using a sonochemical approach is shown in figure 3.

Research by Anandana *et al*. showcased the synthesis of Bi copper oxide nanoparticles via the sonochemical method, demonstrating efficacy in water pollutant removal [107]. Another study reported the fabrication of needle-shaped Bi_2_Mo_3_O_12_·5H_2_O nanostructures via sonochemical synthesis for Rhodamine B dye degradation, highlighting its potential in environmental remediation [108]. A study by Wang *et al*. [109] demonstrated the synthesis of uniform nanorods of Bi sulfate through ultrasound irradiation, with particle diameters ranging from 20 to 30 nm and lengths of 200−250 nm, using sodium thiosulfate and Bi nitrate pentahydrate as precursors. Modifying the precursor to thioacetamide resulted in smaller and narrower nanorods owing to increased nucleation rates. Additionally, altering the chelating agent influenced the growth and nucleation rates, leading to nanorods with varying dimensions. For instance, nanorods synthesized using EDTA as the chelator exhibited diameters of 15 nm and lengths of 100 nm, while those produced with sodium tartrate were smaller and more agglomerated, with average dimensions of 20 × 60 nm. The addition of 20% N,N-dimethylformamide to the solution increased yield and decreased nanorod dimensions to 6 × 30 nm, attributed to accelerated nucleation [109]. These findings underscore the versatility and effectiveness of sonochemistry in producing Bi-based photocatalysts with tailored properties for various applications.

### 2.5. Solvothermal technique

The solvothermal technique, a method using specific solvent mediums under varying pressures (1–10 000 atm) and temperatures (100–1000°C), enables the synthesis of diverse Bi nanostructures [48,110]. This approach requires medium to high pressures (starting from 105 Pa) and temperatures and meanwhile facilitates interactions among starting materials, leading to the formation of nanomaterials with distinct morphologies [111–113]. Notably, Cheng *et al*. [114] used this technique to produce Bi subcarbonate nanostructures in the forms such as cubes and plates, employing Bi nitrate as a precursor and showcasing the influence of precursors and solvents on the resulting morphology. Further advancements include the synthesis of Bi alloy nanoparticles using ethylene glycol as both solvent and reducing agent, revealing that particle size and distribution vary with stabilizing agents and metal salts. Moreover, heterostructures combining BiOCl and titanium oxide were developed that showed enhanced photocatalytic efficiency in degrading methylene blue under sunlight. Using glucose as a reducing agent, Bi molybdate photocatalysts were prepared, exhibiting an Aurivillius-type structure, significant for the removal of heavy metal ions and organic dyes from wastewater. For example, Li *et al*. [115] demonstrated that glucose-assisted hydrothermal synthesis of plasmonic Bi-deposited nested Bi_2_O_2_−xCO_3_ photocatalysts significantly enhances photocatalytic activity, achieving removal efficiencies of 85% for Lanasol red and 88% for ciprofloxacin under simulated solar irradiation, surpassing the performance of control sample. Zhou *et al*. [116] further explored the role of reducing agents in the solvothermal synthesis of Bi-oxide nanostructures, indicating the morphology’s dependence on the presence of electron donors like ascorbic acid, which led to the formation of nanoplates, in contrast to spherical structures obtained in its absence. In summary, the solvothermal technique emerges as a versatile method for synthesizing Bi nanostructures, offering control over morphology through the choice of solvents and precursors. Its use spans photocatalysis and wastewater treatment, highlighting its potential in environmental remediation and the development of efficient, non-toxic photocatalysts.

### 2.6. Green synthesis

The green synthesis of Bi nanoparticles represents a significant shift towards environmentally sustainable nanomaterial production, using plant extracts as biogenic mediators [117]. This method attracts more attention attributed to its eco-friendliness, cost-effectiveness, minimal waste generation and reduced chemical usage, offering a benign alternative to conventional nanoparticle synthesis approaches [118]. Bi-oxide nanoparticles, synthesized through green routes, exhibit lower toxicity and are easier to handle, attributed to the use of non-toxic solvents like ethanol and water for plant extract preparation [119,120]. Plant parts including barks, fruits, peels, flowers, leaves and roots serve as sources of rich bioactive compounds such as flavonoids, saponins and phenol carboxylic acids [121–123], which act as natural antioxidants, stabilizers and reducers in nanoparticle formation [124,125]. This method has facilitated the synthesis of various Bi-based nanoparticles with potential applications in antimicrobial activity and pollution control [126,127]. For instance, *Callistemon viminalis* extracts, rich in alkaloids and flavonoids, were used to synthesize Bi vanadate nanorods [128]. Similarly, Das *et al*. [129] employed hydroalcoholic extracts from *Moringa oleifera* leaves, containing polyphenols, to reduce Bi (III) ions to elemental Bi nanoparticles. Furthermore, nanoparticles synthesized from *Eclipta alba* and *Nephelium lappaceum* peels have demonstrated significant microbial control, suggesting their use in public sanitation [130]. Additionally, non-plant biological molecules like human and bovine serum albumin, along with gelatin, have been explored for Bi nanoparticle synthesis, implying the versatility and breadth of green synthesis methods [131]. These eco-conscious strategies not only align with principles of green chemistry but also open new pathways for producing tailored nanoparticles with controlled sizes and shapes, expanding their applicability across various domains, including catalysis and environmental remediation.

### 2.7. Laser-aided technique

The laser-aided technique for synthesizing Bi-based photocatalysts involves using laser irradiation to induce reactions allowing the formation of Bi nanoparticles or structures [132–134]. This method stands out for its precision, reaction rate and ability to control the size, morphology and crystallinity of the photocatalysts without the need for high temperatures or toxic chemicals [135–138]. In this process, a laser beam is directed at a target material containing Bi, often in a liquid medium (figure 3c). The laser’s energy causes rapid localized heating, leading to the evaporation of the target material and its subsequent condensation into nanoparticles. The parameters of the laser (such as wavelength, pulse duration and power) and the environment (such as the type of solvent, presence of stabilizers and atmosphere) are crucial in determining the characteristics of the resulting photocatalyst [139,140]. Laser synthesis can be performed in either a pulsed or continuous-wave mode, affecting the energy delivery and, consequently, the reaction dynamics [135–138]. Pulsed lasers allow for high peak powers without thermal damage to the surrounding material, promoting the production of nanoparticles with narrow size distributions and high purity [141,142]. Continuous-wave lasers, on the other hand, can be used for the continuous production of materials but may require better thermal management [143,144]. This method offers precise control over the size and morphology of nanoparticles by adjusting laser parameters, including pulse duration, energy, repetition rate and wavelength [145]. Furthermore, altering the pH conditions in the liquid medium facilitates the creation of diverse nanostructures, such as hollow spheres and core–shell configurations, showcasing the technique’s versatility in tailoring Bi nanomaterials for advanced photocatalytic applications. This technique is highly valued for its ability to produce photocatalysts with specific surface properties, enhanced active sites and tailored band gaps, which are critical for optimizing the photocatalytic degradation of pollutants, including MC-LR. The precision and control offered by laser-aided synthesis make it an attractive approach for advancing Bi photocatalyst technology.

### 2.8. Synthesis of monodispersed bismuth-oxide nanoparticles in solution

The synthesis of highly monodispersed Bi-oxide nanoparticles with tuneable size and shape in solution, without the need for high-pressure conditions, is of significant interest to colloid chemists and researchers in nanoparticle science. Several solution-based methods have been developed to achieve this, including the sol–gel method, chemical reduction, microwave-assisted synthesis and green synthesis. The sol–gel method involves the hydrolysis and condensation of Bi precursors (e.g. Bi nitrate) in a solvent, followed by controlled gelation and calcination, yielding nanoparticles with sizes ranging from 20 to 100 nm, as demonstrated by Wang *et al*. [100]. The chemical reduction method employs reducing agents such as sodium borohydride or hydrazine to reduce Bi precursors in the presence of stabilizing agents, producing nanoparticles with sizes between 10 and 50 nm [85]. Microwave-assisted synthesis offers rapid and uniform heating, enabling the formation of monodispersed Bi-oxide nanoparticles (10–80 nm) with controlled size and shape, as shown by Li *et al*. [73]. Additionally, green synthesis uses plant extracts (e.g. *Mo. oleifera*) as natural reducing and stabilizing agents, producing eco-friendly, colloidally stable nanoparticles (20–60 nm) [129]. These methods provide precise control over nanoparticle properties, making them highly relevant for applications in colloid chemistry and advanced material science.

## 3. Mechanism of MC-LR degradation

The adsorption of MC-LR on the surface of Bi-based photocatalysts is governed by several interactions, including electrostatic forces, hydrogen bonding, π–π stacking, hydrophobic interactions and surface complexation. These interactions are influenced by the surface properties of the photocatalyst (e.g. surface charge and functional groups) and the protonation state of MC-LR, which varies with pH. For example, at acidic pH, MC-LR is positively charged owing to the protonation of amino groups, facilitating its adsorption on negatively charged Bi oxyhalide surfaces through electrostatic attraction. Hydrogen bonding between the polar groups of MC-LR and the surface hydroxyl or halide ions further enhances adsorption. Additionally, π–π stacking and hydrophobic interactions between the aromatic Adda side chain of MC-LR and the layered structure of Bi oxyhalides contribute to adsorption. These interactions not only facilitate the adsorption of MC-LR but also position it closer to the active sites, enhancing its exposure to ROS and accelerating its degradation.

Photocatalytic technology uses semiconductor photocatalysts under light to produce highly active radicals (e.g. ·OH, ·O_2_^−^, 1O_2_ and H_2_O_2_) [146,147]. These radicals affect algae cells’ physiological processes, inhibiting and killing them. For example, Bi_2_O_3_’s mechanism can be expressed by the reaction formula presented in equations (3.1)–(3.6)


(3.1) $$Bi_{2}O_{3}+h\nu \to h^{+}+e^{-},$$

(3.2) $$h^{+}+H_{2}O\to \;\cdot OH+H^{+},$$

(3.3) $$e^{-}+O_{2}\to \;\cdot O_{2}^{-},$$

(3.4) $$\cdot O_{2}^{-}+H^{+}\to HOO\cdot ,$$

(3.5) $$e^{-}+HOO\cdot +\;H^{+}\to H_{2}O_{2},$$

(3.6) $$H_{2}O_{2}+e^{-}\to \;\cdot OH+OH^{-}.$$

Based on this basic principle, most of the research has explained the photocatalytic mechanisms for the Bi-photocatalyst-assisted degradation of MC-LR. The effectiveness of photocatalysis hinges on factors like surface area, phase structure and charge transfer efficiency [148]. Herein, the surface area plays a crucial role by providing more active sites for the adsorption of pollutants and facilitating photocatalytic reactions. The phase structure, referring to the crystalline arrangement of the material, influences its electronic properties, charge separation capabilities and overall photocatalytic performance. Moreover, efficient charge transfer processes are essential, as they determine how effectively photogenerated electrons and holes migrate to the material’s surface to participate in redox reactions, thereby reducing recombination and enhancing photocatalytic efficiency. Optimization of these factors is critical in developing high-performance Bi-photocatalysts for environmental remediation and other applications. Composite Bi-photocatalysts promise to improve charge separation, increase carrier lifetimes and enhance interfacial charge transfer efficiency [149]. It is worth mentioning that the grain boundaries, trap states and surface states in Bi-based photocatalysts significantly influence the charge carrier separation processes. Grain boundaries can act as recombination centres but can also create electric fields that facilitate charge separation when properly engineered. Trap states, particularly shallow ones, can temporarily capture electrons or holes, reducing recombination rates and enhancing photocatalytic activity. Surface states, on the other hand, play a dual role: they can act as recombination centres or facilitate charge transfer to adsorbed species, such as water or oxygen molecules, leading to the generation of ROS. Additionally, the Bohr radius of excitons, which determines the average distance between an electron and a hole, is a critical factor in charge separation. In Bi-based photocatalysts, the exciton Bohr radius is influenced by the material’s dielectric constant and effective mass of charge carriers. A smaller Bohr radius typically leads to stronger electron–hole binding, making charge separation more challenging. However, nanostructuring or doping can reduce the exciton binding energy, facilitating charge separation and improving photocatalytic performance.

**Figure 4.**
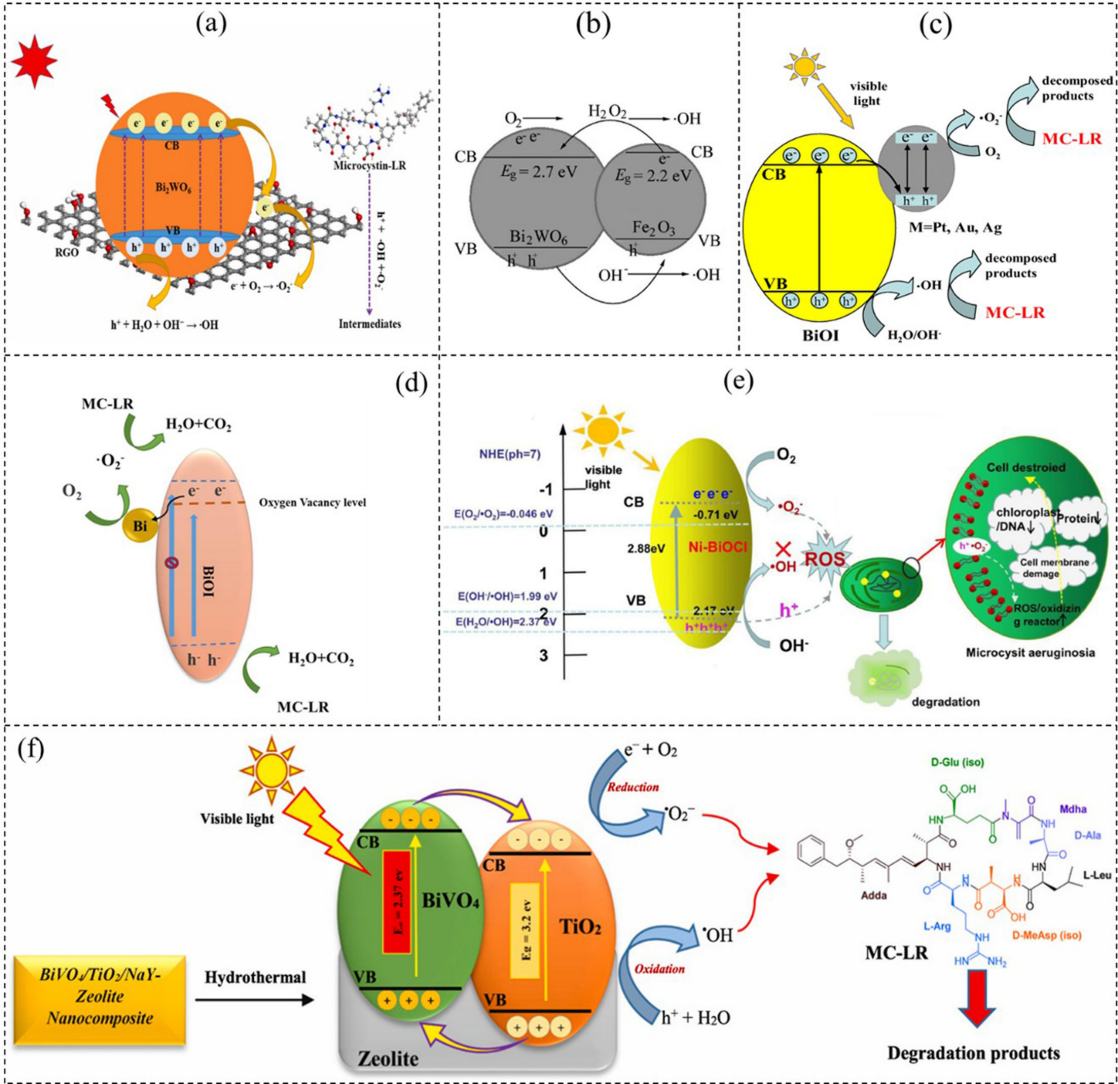
(a) The photocatalytic mechanism for MC-LR degradation over Bi_2_WO_6_/RGO_3_% (reprinted with permission [33], copyright, Elsevier 2023). (b) Band structure and charge migration process of Fe_2_O_3_/Bi_2_WO_6_ (reprinted with permission [152], copyright, Elsevier 2021). (c) Precious metal-loaded BiOI semiconductor for photocatalytic degradation of MC-LR (reprinted with permission [153], copyright, Springer 2019). (d) The mechanism involved in MC-LR degradation using x-BiOI nanocomposite (reprinted with permission [28], copyright, Springer 2020). (e) The photocatalytic degradation organics of MC-LR by Ni-BiOCl % (reprinted with permission [154], copyright, Elsevier 2020). (f) The photocatalytic mechanism for MC-LR degradation over Bi_2_WO_6_/RGO3% (reprinted with permission [155], copyright, Elsevier 2020).


Lin *et al*. [150] investigated the Fe_2_O_3_/Bi_2_WO_6_ composite for the photocatalytic degradation of MC-LR. They found that the composite exhibited enhanced photocatalytic activity attributed to its ability to absorb a broader range of visible light and effectively inhibit charge recombination. To understand this enhanced activity, they analysed the relative band positions of Fe_2_O_3_ and Bi_2_WO_6_. Bi_2_WO_6_’s conduction band edge was found to be lower than that of Fe_2_O_3_, enabling an irreversible transfer of carriers at the interface between the two semiconductors. Under visible-light irradiation, both Fe_2_O_3_ and Bi_2_WO_6_ generated photoinduced electrons and holes. Owing to the internal electric field, some electrons in the conduction band of Fe_2_O_3_ were quickly injected into the conduction band of Bi_2_WO_6_, increasing the separation of photogenerated electron–hole pairs [151]. Simultaneously, holes in the valence band of Bi_2_WO_6_ could migrate to the valence band of Fe_2_O_3_. This charge transfer process increased the availability of electrons to O_2_ and further facilitated the generation of H_2_O_2_ and then immediately converted to ·OH. MC-LR can be broken into smaller molecules by generating potent oxidizing agents such as H_2_O_2_ and ·OH. The study provides detailed insights into the mechanism underlying the enhanced photocatalytic activity of the Fe_2_O_3_/Bi_2_WO_6_ composite for MC-LR degradation. Based on Lin *et al*. [150], the possible photocatalysis mechanism of this composite Bi-photocatalyst (figure 4b) is shown in equations (3.7)–(3.13)

(3.7) $$Fe_{2}O_{3}/Bi_{2}WO_{6}+h\nu \to Fe_{2}O_{3}\left(e^{-}+h^{+}\right)/\;Bi_{2}WO_{6}\left(e^{-}+h^{+}\right),$$

(3.8) $$Fe_{2}O_{3}\left(h^{-}+h^{+}\right)/Bi_{2}WO_{6}\left(h^{-}+h^{+}\right)\to Fe_{2}O_{3}\left(h^{+}\right)+Bi_{2}WO_{6}\left(h^{+}\right),$$

(3.9) $$e^{-}+O_{2}\to \cdot O_{2}^{-},$$

(3.10) $$e^{-}+\cdot O_{2}^{-}+2H^{+}\to H_{2}O_{2},$$

(3.11) $$H_{2}O_{2}+e^{-}\to OH^{-}+\cdot OH,$$

(3.12) $$h^{+}+H_{2}O\to \cdot OH+H^{+},$$

(3.13) $$h^{+}+\cdot OH+MC\text{-}LR\to \text{products}.$$

Zhan *et al*. [33] investigated the visible-light-driven photocatalytic degradation of MC-LR using Bi_2_WO_6_/reduced graphene oxide (RGO) heterojunctions. They confirmed the presence of active species (·OH and ·O_2_^−^) through electron spin resonance spectroscopy. Under dark conditions, no peak of DMPO–∙OH or DMPO–∙O_2_^−^ was detected, indicating that these species were not present (figure 4a). However, in the presence of visible light, spectrum peaks corresponding to DMPO–∙OH and DMPO–∙O_2_^−^ were observed, confirming their presence. This demonstrated that h^+^, ·OH and ·O_2_^−^ were the species attributed to MC-LR degradation. The proposed mechanism involves the generation of electron–hole pairs under visible light, with photogenerated electrons moving to RGO to produce ·O_2_^−^, while h^+^ directly assists the MC-LR degradation in the presence of ·OH and ·O_2_^−^ (equations (3.14)–(3.18)):


(3.14) $$MC\text{-}LR+Bi_{2}WO_{6}/RGO\to Bi_{2}WO_{6}/RGO\text{-}MC\text{-}LR_{\left(adsorbed\right)},$$

(3.15) $$Bi_{2}WO_{6}/RGO\text{-}MC\text{-}LR+h\nu \to Bi_{2}WO_{6}/RGO\left(e^{-}\right)+Bi_{2}WO_{6}/RGO\left(h^{+}\right),$$

(3.16) $$h^{+}+H_{2}O\to \cdot OH+H^{+},$$

(3.17) $$e^{-}+O_{2}\to \cdot O_{2}^{-},$$

(3.18) $$MC\text{-}LR+h^{+}+\cdot OH+\cdot O_{2}^{-}\to \text{by-products}.$$

Lin *et al*. [150] investigated the degradation mechanism of MC-LR by Bi_2_WO_6_/ZnO/biochar composites. They conducted radical trapping experiments using various scavengers to elucidate the role of different reactive species in the photocatalytic degradation process. Isopropanol and p-benzoquinone, scavengers for hydroxyl radicals (·OH) and superoxide radicals (·O_2_^−^), respectively, were found to inhibit the degradation of MC-LR, indicating that ·OH and ·O_2_^−^ were the main active species responsible for the degradation process. On the other hand, sodium thiosulfate and sodium oxalate, which act as scavengers for electrons (e^−^) and holes (h^+^), respectively, did not affect the degradation rate of MC-LR. This suggests that e^−^ and h^+^ were not the dominant active species involved in the degradation process. The proposed mechanism involves the excitation of electrons from the valence band to the conduction band of Bi_2_WO_6_ under visible light, creating a hole near the valence band. These electrons then migrate to the ZnO component of the composite, where they capture oxygen molecules to form ·O_2_^−^. The ·O_2_^−^ ions generated in the valence band of ZnO oxidize water molecules to produce ·OH radicals, which are responsible for the degradation of MC-LR adsorbed on the Bi_2_WO_6_ surface. The entire mechanism is presented in equations (3.19)–(3.24). The biochar component derived from rice husks provides a mesoporous structure, increasing the specific surface area of the ZnO photocatalyst and providing more active sites for the photocatalytic reaction. Additionally, the heterostructure formed between Bi_2_WO_6_ and ZnO reduces the recombination probability of electron–hole pairs, leading to enhanced photocatalytic activity. Analyses using techniques like transmission electron microscopy and X-ray photoelectron spectroscopy (XPS) confirmed the heterogeneous structure of the composite and its correlation with photocatalytic efficiency


(3.19) $$Bi_{2}WO_{6}+h\nu \to Bi_{2}WO_{6}\left(e^{-}+h^{+}\right),$$

(3.20) $$Bi_{2}WO_{6}\left(e^{-}\right)+ZnO\to Bi_{2}WO_{6}+ZnO\left(e^{-}\right),$$

(3.21) $$ZnO\left(e^{-}\right)+O_{2}\to ZnO+\cdot O_{2}^{-},$$

(3.22) $$Bi_{2}WO_{6}\left(h^{+}\right)+H_{2}O\to \cdot OH+H^{+},$$

(3.23) $$\cdot O_{2}^{-}+MC\text{-}LR\to \text{degradation products}\to CO_{2}+H_{2}O,$$

(3.24) $$\cdot OH+MC\text{-}LR\to \text{degradation products}\to CO_{2}+H_{2}O.$$

Bi-oxide/metal heterostructures, such as Bi_2_O_3_/Au or BiOI/Ag, have shown significant promise in enhancing photocatalytic activity owing to the role of metal domains as electron sinks [156]. The lower Fermi level of the metal compared to the conduction band of the semiconductor facilitates the transfer of photogenerated electrons from the semiconductor to the metal, reducing electron–hole recombination and enhancing charge separation. This process is further supported by the formation of a Schottky barrier at the Bi-oxide/metal interface, which promotes the accumulation of electrons on the metal domains and holes on the semiconductor surface. Additionally, metal domains can exhibit surface plasmon resonance, which enhances light absorption and generates hot electrons that can be transferred to the conduction band of the semiconductor, further improving photocatalytic activity. For example, Bi_2_O_3_/Au heterostructures have been shown to significantly enhance the degradation of MC-LR under visible light, with the Au domains acting as efficient electron sinks and promoting the generation of ROS. These findings highlight the potential of Bi-oxide/metal heterostructures in photocatalytic applications for water treatment.

Xu *et al*. [157] created a van der Waals heterojunction photocatalyst using BiO_2_−x coupled with Bi_3_NbO_7_ for the inactivation of *Microcystis aeruginosa*. ·O_2_^−^ from the photoexcitation process damaged algae antioxidant systems and cell membranes, releasing degradable organic matter and MC-LR followed by algae death. Additionally, BiO_2_−x/Bi_3_NbO_7_ effectively degraded organic matter, suggesting promising pathways for MC-LR degradation. Feng *et al*. [154] investigated the degradation performance of Ni-doped BiOCl photocatalytic materials, finding that 9% Ni-BiOCl showed efficient degradation of inorganic substances (rhodamine b and bisphenol a) and microorganisms (*Mi. aeruginosa*) under visible light. The mechanism involved the generation of ·O_2_^−^ and h^+^ species, which damaged algae cell walls and inactivated intracellular substances, leading to further degradation into inorganic compounds. Depending on the specific degradation pathway, the final products could include carbon dioxide (CO_2_), water (H_2_O) and inorganic salts. Zhang *et al*. [153] constructed precious metal-loaded BiOI semiconductor materials for enhanced photocatalytic degradation of MC-LR. Tu *et al*. [158] developed a floating integral structure using agricultural waste loofah sponge loaded with rGO/BiOBr for treating algae-contaminated wastewater. This composite showed significant photocatalytic activity in inactivating *Mi. aeruginosa* by producing ·O_2_^−^ and h^+^ that disrupted cell structures and membranes, causing cytoplasmic content leakage and cell degradation.

Yanfen *et al*. [159] conducted a study focusing on the unique ability of BiOBr to selectively decarboxylate d-glutamic acid (Glu) and methyl-d-aspartic acid (d-MeAsp) in the photocatalytic degradation of MC-LR in water. They explored the mechanism and found that BiOBr produced superoxide radicals (·O_2_^−^) and holes (h^+^) under visible light, which played a crucial role in degrading MC-LR. The photocatalytic degradation process involves several steps that result in the breakdown of MC-LR molecules (equations (3.25)–(3.28)). One of the key findings of the study is the selective decarboxylation of d-Glu and d-MeAsp in the presence of BiOBr. This decarboxylation occurs before the collapse of the macro ring structure of MC-LR, indicating a unique feature of BiOBr photocatalysis compared to traditional TiO_2_ photocatalysis. In TiO_2_ photocatalysis, the primary reaction is dominated by positive holes and does not result in the decarboxylation of free acid groups during the degradation of MC-LR in water. This is because TiO_2_ surface holes have a high oxidation potential, leading to the oxidation of adsorbed water and the generation of hydroxyl radicals rather than directly oxidizing the carboxylate groups of MC-LR. By contrast, BiOBr’s valence band, consisting of a mixture of O 2p orbitals and Br 4p orbitals, along with the conduction band, consisting mainly Bi 6p orbitals, together allow for the selective oxidation of the carboxylate groups of MC-LR. The valence band of BiOBr facilitates the coordination of the protonated free acid group of MC-LR through hydrogen bond interaction, leading to the direct reaction of photoinduced holes with the carboxylate groups equation (3.29). This process results in the selective decarboxylation of amino acids in MC-LR, a crucial step in its degradation. Furthermore, the study used ^18^O from H_2_^18^O as a signature label by using H_2_^18^O as the reaction matrix to investigate the MC-LR degradation pathway. This incorporation indicates the following two possible pathways: direct oxidation of MC-LR to generate an oxidative intermediate equation (3.30) or formation of a positive ion radical fragment of MC-LR followed by the capture of ^16^O_2_ to form a ketone product containing ^16^O equation (3.31). These pathways demonstrate the complex nature of MC-LR degradation and highlight the need for further research to identify detailed reaction intermediates


(3.25) $$RCONHCH\left(R\right)COOH-e^{-}\to {\left[RCONHCH\left(R\right)COOH\right]}^{\cdot +}\to RCONHCH\left(R\right)COO^{\cdot }+H^{+},$$

(3.26) $$RCONHCH\left(R\right)COO^{\cdot }\to RCONH^{\cdot }CH\left(R\right)+CO_{2},$$

(3.27) $$RCONH^{\cdot }CH\left(R\right)+RCONHCH\left(R\right)COOH\to RCONHCH_{2}\left(R\right)+RCONH^{\cdot }C\left(R\right)COOH,$$

(3.28) $$RCONHCH_{2}CH\left(R\right)COOH+\cdot OH\;radical\to RCONHCH_{2}^{\cdot }C\left(R\right)COOH+H_{2}O,$$

(3.29) $$\left(MC\text{-}LR\right)\text{-}COOH+h^{+}\left(\text{BiOBr}\right)\to {\left[{\left(MC\text{-}LR\right)}_{ad}\text{-}COOH\right]}^{\cdot +}\to \text{fragment}{\left(MC\text{-}LR\right)}^{+\cdot }+CO_{2},$$

(3.30) $$\text{fragment}{\left(MC\text{-}LR\right)}^{\cdot }+H_{2}^{18}O\to \text{fragment}\left(MC\text{-}LR\right){\text{-}}^{18}OH+H^{+}\to {\text{ketone-}}^{18}O,$$

(3.31) $$\text{fragment}{\left(MC\text{-}LR\right)}^{+\cdot }{+}^{16}O_{2}\to \text{fragment}\left(MC\text{-}LR\right)\text{-}OO^{\cdot }\to {\text{ketone-}}^{16}O.$$

In our conclusion, the mechanism of photocatalytic algae and algal toxins inhibition by Bi-based materials involves several key aspects. Firstly, nanomaterials adsorb onto algae cell membranes through electrostatic interactions, disrupting their normal metabolic processes. Secondly, these materials inhibit photosynthesis by damaging the photosynthetic system and degrading chlorophyll a, thereby inhibiting enzyme activities. Thirdly, they alter cell membrane composition, leading to structural damage and content leakage. Lastly, ROS accumulate within algal cells, causing lipid peroxidation, structural damage and the release of intracellular ions, ultimately leading to cell dissolution.

## 4. MC-LR degradation pathway

**Figure 5.**
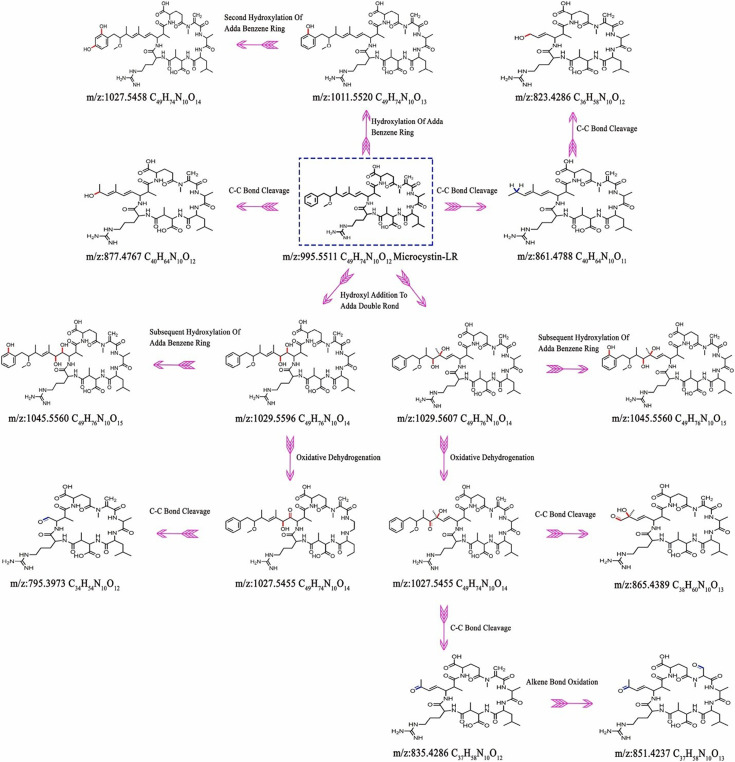
MC-LR degradation pathways using Bi_2_WO_6_/RGO heterojunctions under visible light (reprinted with permission [33]. Copyright, Elsevier 2023).

Study of the MC-LR degradation pathway is crucial to understanding the cleavage location and ensuring the complete remediation of toxicity or no toxic intermediates formed during the degradation. This information will assist in optimizing the photocatalytic conditions. Zhan *et al*. [33] investigated MC-LR degradation pathways using Bi_2_WO_6_/RGO heterojunctions under visible light. Liquid chromatography with tandem mass spectrometry (LC–MS/MS) analysis revealed 11 intermediates, including those resulting from cleavage of the C8–C9 and C6–C7 bonds in the Adda side chain, and hydroxylation of the Adda benzene ring, with *m*/*z* values of 877.4767, 861.4788, 823.4286, 1011.5520 and 1027.5458 (figure 5). These findings provide insight into the mechanism of MC-LR degradation by Bi_2_WO_6_/RGO 3%, showing promise for applying this photocatalyst in water treatment. The study by Wang *et al*. [35] revealed the formation of intermediates at *m*/*z* 1029.5596 and 1029.5607 through the hydroxylation of MC-LR by ∙OH attacked on the C4–C5 or C6–C7 double bonds of the Adda side chain. These intermediates further transformed into *m*/*z* 1045.5560 and *m*/*z* 1027.5455 through additional hydroxylation and oxidative dehydrogenation, respectively. The *m*/*z* 1027.5455 intermediate led to the formation of *m*/*z* 795.3973 by cleaving the C4–C5 bond. Additionally, the *m*/*z* 1029.5596 intermediate could form *m*/*z* 1027.5455, which further produced *m*/*z* 865.4389 by cleaving the C7–C8 bond. The product at *m*/*z* 835.4286 resulted from the C6–C7 bond cleavage in the Adda chain and indicates the degradation of MC-LR and the reduction of its toxicity during the photocatalytic process. The oxidation of the alkene bond in the Mdha chain, converting *m*/*z* 835.4286 to *m*/*z* 851.4237, was also observed in a previous study using BiOBr photocatalysis. These findings were cross-checked with studies done by other groups and found consistent [35,160].

**Figure 6.**
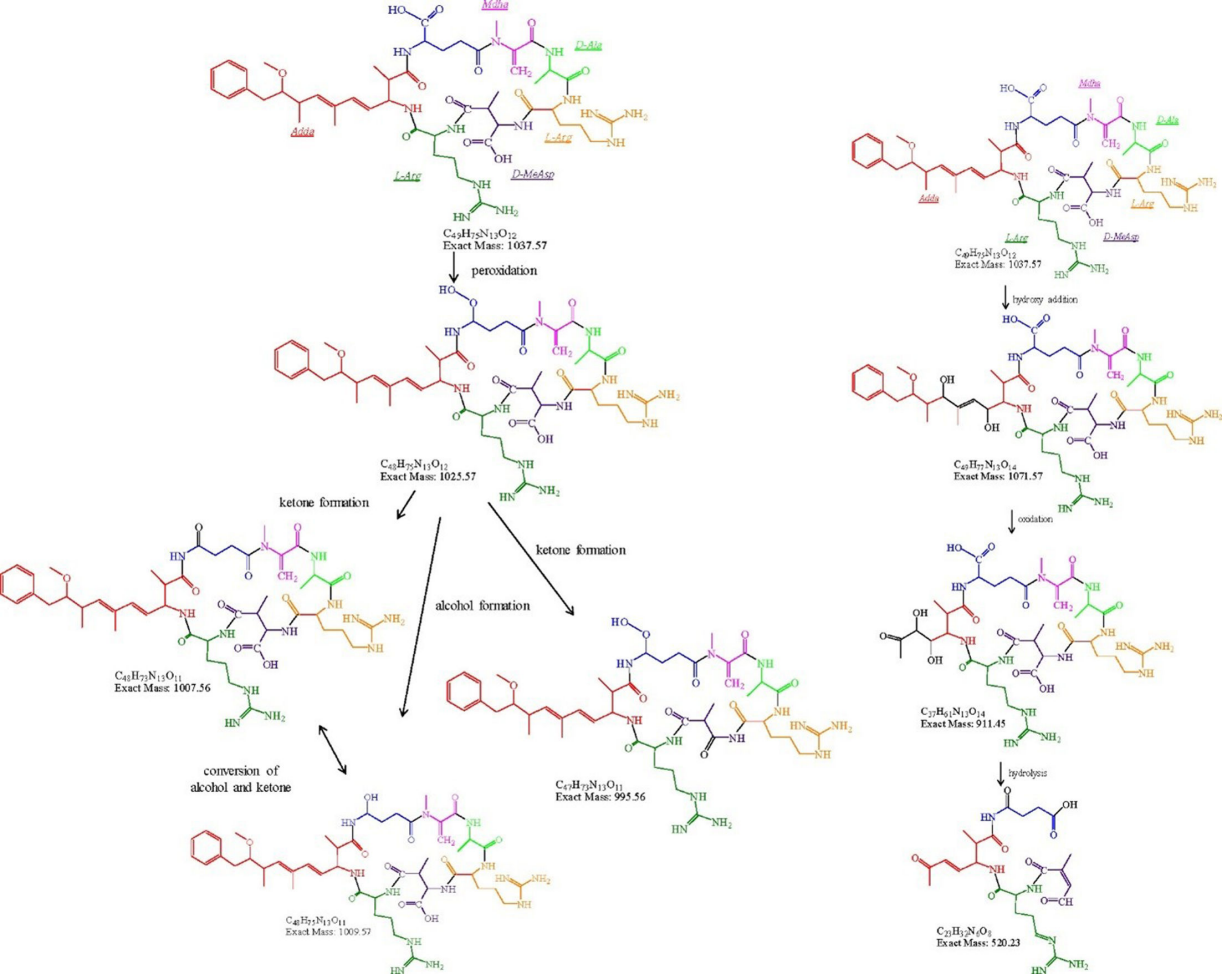
The degradation pathways of MC-RR over Ag/Ag_2_O-BiVO_4_ under visible-light irradiation (reprinted with permission [161]. Copyright, Elsevier 2017).

This pathway is also applicable to the other analogue of MC like microcystin-arginine(R)-argining (R) (MC-RR), which differs from MC-LR by the amino acid in position Y on the heptapeptide ring, whereas MC-LR has leucine (L), but MC-RR has arginine (R). For example, Wu *et al*. [161] studied the degradation pathway of MC-RR by exposing it to an Ag/Ag_2_O-BiVO_4_ catalyst under visible-light irradiation. LC-MS analysis detected intermediates formed during the photodegradation process. Two degradation pathways were proposed (figure 6): peroxidation and hydroxylation. In the peroxidation pathway, a peroxidation intermediate (*m*/*z* = 1025.57) is formed first, probably owing to decarboxylation by photogenerated holes, followed by transformation into ketones or hydroxylated intermediates (*m*/*z* = 1009.57, 995.56 and 1007.56). The hydroxylation pathway involves attacks on unsaturated double bonds in the Adda residue by ∙OH, leading to hydroxylated intermediates (*m*/*z* = 1071.57). Cleavage of these bonds generates an intermediate (*m*/*z* = 911.45) that can further hydrolyse into other products, suggesting the initial photodegradation of MC-RR occurs through both pathways.

Based on the understanding of the MC-LR photocatalysis mechanism discussed above, the degradation pathway can be summarized by a multi-step process, leading to the breakdown of the MC-LR molecule into smaller, less toxic compounds. Here is a general outline of the possible degradation pathway: (i) the MC-LR molecule adsorbs onto the surface of the photocatalyst, facilitating its interaction with reactive species generated during photocatalysis; (ii) upon exposure to light, the photocatalyst generates ROS (such as •OH and •O_2_^−^) through photocatalytic reactions; (iii) the generated ROS attacks the MC-LR molecule, oxidizing its peptide bonds and other functional groups. This results in the cleavage of the peptide ring and the formation of smaller fragments; (iv) further oxidation and cleavage reactions lead to the fragmentation of the MC-LR molecule into smaller peptides and amino acids; (v) the smaller fragments undergo further oxidation and degradation, eventually leading to the complete mineralization of MC-LR into inorganic compounds such as CO_2_, H_2_O and mineral salts; and (vi) throughout the degradation process, various intermediate by-products may be formed, including shorter peptides, amino acids and other organic compounds. These intermediates are typically less toxic than MC-LR and can undergo further degradation or be mineralized. Overall, the photocatalytic degradation of MC-LR involves a series of oxidative reactions facilitated by ROS generated by the photocatalyst. The process ultimately leads to the breakdown of MC-LR into non-toxic or less toxic compounds, making it a promising approach for water treatment applications.

## 5. Prospects and challenges

The recyclability, reusability and compositional stability of Bi-based photocatalysts are crucial for their practical application. Studies have shown that many Bi-based photocatalysts, such as Bi₂WO₆, BiOI and BiOCl, exhibit excellent recyclability and retain high photocatalytic activity over multiple cycles. For example, Zhang *et al*. [153] demonstrated that a precious metal-loaded BiOI photocatalyst maintained over 90% of its activity after five cycles of MC-LR degradation. Similarly, Feng *et al*. [154] reported that Ni-doped BiOCl showed minimal loss of activity after four cycles. Structural and compositional stability, confirmed through X-Ray Diffraction (XRD) and X-Ray Photoelectron Spectroscopy (XPS) analysis, further supports the robustness of these materials. However, challenges such as surface fouling and slight leaching of Bi ions may occur, necessitating strategies like surface modification or immobilization to enhance long-term stability [162].

Based on the previous literature and the above discussion, we proposed some prospects and challenges of Bi-based photocatalysts in MC-LR degradation. The challenges include the following:

(i) Bi-based photocatalysts may suffer low efficiency in degrading MC-LR owing to limited light absorption, rapid recombination of photogenerated charge carriers and insufficient catalytic active sites;
(ii) some Bi-based photocatalysts may exhibit poor stability, leading to degradation or agglomeration over time. This can result in a decrease in catalytic activity and a shortened catalyst lifespan;
(iii) Bi-based photocatalysts may exhibit selectivity in degrading specific components of MC-LR, leading to the formation of harmful by-products or incomplete degradation of MC-LR molecules; and
(iv) finding the optimal conditions for Bi-based photocatalysts to achieve maximum MC-LR degradation can be challenging. Factors such as catalyst loading, light intensity, pH and temperature must be carefully optimized.

The prospects include the following:

(i) researchers are exploring ways to enhance the photocatalytic activity of Bi-based materials. This includes doping with other elements, such as nitrogen or sulfur, which can improve the separation of photogenerated charge carriers and enhance catalytic performance;
(ii) developing Bi-based photocatalysts with enhanced selectivity towards MC-LR degradation over other organic pollutants is a key prospect. This can be accomplished by modifying the catalyst’s surface or designing its structure to specifically target the functional groups within the MC-LR molecule;
(iii) scaling up the production of Bi-based photocatalysts and testing their performance in field and engineering applications is a significant prospect. This includes assessing their effectiveness in large-scale water treatment plants and their compatibility with existing treatment processes; and
(iv) assessing the environmental impact of Bi-based photocatalysts is crucial. This includes evaluating their long-term stability, toxicity and potential for bioaccumulation to ensure their safety and sustainability for water treatment applications.

In summary, the highlighted key challenges and future directions in using Bi-based photocatalysts for MC-LR degradation are as follows: (i) while there is a robust theoretical basis for using photocatalysts in laboratories, practical applications for controlling harmful algal blooms are limited. Future research should focus on practical applications and expanding the use of bio-based materials to degrade algal toxins; (ii) while modification of Bi-based materials is advancing, methods remain relatively simple. Future studies should explore complex composite modifications to enhance the response to visible light; and (iii) most bio-based materials are powdered, making recovery challenging. Future research work may explore viable ways to immobilize Bi-based semiconductors onto floatable materials for enhanced solar light absorption and minimize waste generation through convenient catalyst retrieval in field application.

## 6. Conclusion

Bi-based photocatalysts show great promise for the degradation of MC-LR and other organic contaminants in water bodies. Their unique properties, including visible-light response, stability and low toxicity, make them attractive candidates for water treatment applications. However, several challenges remain to be addressed to fully exploit the potential of Bi-based photocatalysts for MC-LR degradation. Future research should focus on further enhancing the photocatalytic activity of Bi-based photocatalysts through the optimization of their composition, morphology and doping strategies. Additionally, the mechanisms of MC-LR degradation on Bi-based photocatalysts should be further elucidated to improve the understanding of the degradation process. Overall, Bi-based photocatalysts hold great promise for the degradation of MC-LR and other organic contaminants in water bodies. With continued research and development, these photocatalysts could play a crucial role in ensuring the safety and sustainability of our water resources.

## Data Availability

This article has no additional data.
